# Draft Genome Sequence of *Actinobaculum massiliense* Strain FC3

**DOI:** 10.1128/genomeA.01537-15

**Published:** 2016-01-14

**Authors:** Mamadou Beye, Sofiane Bakour, Noémie Labas, Didier Raoult, Pierre-Edouard Fournier

**Affiliations:** a Unité de recherche sur les maladies infectieuses et tropicales émergentes (URMITE), UM 63, CNRS 7278, INSERM 1095, IRD 198, IHU Méditerranée Infection, Faculté de Médecine, Aix-Marseille-Université, Marseille, France; b Special Infectious Agents Unit, King Fahd Medical Research Center, King Abdul Aziz University, Jeddah, Saudi Arabia

## Abstract

*Actinobaculum massiliense* strain FC3 was isolated from the urine of a patient with acute cystitis. The 2.06-Mb genome of strain FC3 contains 17 toxin/antitoxin modules and 9 bacteriocin-encoding genes that may play a role in virulence. The genome also exhibits 693 genes acquired by lateral gene transfer.

## GENOME ANNOUNCEMENT

The genus *Actinobaculum* (Lawson, 1997) was created in 1997 by reclassifying *Actinomyces suis*, a bacterium known to cause metritis in pigs (1). According to the List of Prokaryotic Names with Standing in Nomenclature website, the genus *Actinobaculum* currently contains 4 species, including *A. massiliense* (Greub and Raoult, 2006), *A. schaalii* (Lawson et al., 1997), *A. suis* (Wegienek and Reddy, 1982), and *A. urinale* (Hall et al., 2003) (http://www.bacterio.net/actinobaculum.html). The species *Actinobaculum massiliense* was created in 2006. The type strain DSM19118^T^ was isolated from the urine of an elderly woman with recurrent cystitis (2). This species has also been associated with human skin infection (3) and bacteremia (4). It is a Gram-positive, nonmotile, non-acid-fast, and facultatively anaerobic bacterium (2). Here, we report the draft genome sequence of *A. massiliense* strain FC3 (= CSUR P1982 = DSM 100580) that was isolated from the urine of a patient with acute cystitis.

Genomic DNA (gDNA) of *A. massiliense* strain FC3 was sequenced on a MiSeq sequencer (Illumina Inc., San Diego, CA, USA) using the mate-pair strategy. gDNA was quantified by a Qubit assay with the high-sensitivity kit (Life Technologies, Carlsbad, CA, USA) at 54.85 ng/µl. The 1,132,181 paired reads were trimmed and assembled using Spades software (http://bioinf.spbau.ru/spades). Noncoding genes and miscellaneous features were predicted using RNAmmer (5) and Pfam (6), while coding DNA sequences (CDSs) were predicted using Prodigal (7). Functional annotation was achieved using the RAST server (8) and BLAST+ (9) against the COG database.

The genome from *A. massilliense* strain FC3 is 2,065,184-bp long with a 60.17% G+C content. After assembly, the genome is composed of 4 scaffolds. Overall, 1,772 protein-coding genes (1,398 functional and 374 hypothetical proteins) and 60 RNAs (4 complete rRNA operons, 1 additional 5S rRNA, and 47 tRNAs) were identified. There are a total of 1,295 genes (73.08%) assigned a putative function by COGs, and 1,587 genes with Pfam-A domains. Strain FC3 has many genes that are generally related to virulence, including 9 bacteriocin-encoding genes and 17 toxin/antitoxin modules. In contrast, strain FC3 does not contain any genes associated with antibiotic resistance and is devoid of clustered regularly interspaced short palindromic repeats (CRISPRs). Six hundred ninety-three genes are associated with mobile genetic elements, including 213 and 82 genes associated with integrating conjugative elements (ICEs) and phages, respectively. Among these 693 genes, 679 are already listed in the ACLAME database (10).

Hence the genome of strain FC3 contains a large number of genes acquired by lateral gene transfer and several putative virulence markers that are similar to those of *A. schaalii*, which is a recognized uropathogen (1).

### Nucleotide sequence accession numbers.

The 16S rRNA and the genome sequence from *Actinobaculum massiliense* strain FC3 are deposited in GenBank under the accession numbers LN870313 and CYUL01000001 to CYUL01000005, respectively.
